# Association between Childhood Onset Inflammatory Bowel Disease and Psychiatric Comorbidities in Adulthood

**DOI:** 10.3390/diagnostics13111868

**Published:** 2023-05-26

**Authors:** Andreea Sălcudean, Andreea Georgiana Nan, Cristina Raluca Bodo, Marius Cătălin Cosma, Elena Gabriela Strete, Maria Melania Lica

**Affiliations:** 1 Department M2, Discipline of Bioethics, Social & Human Sciences, University of Medicine and Pharmacy, Sciences and Technology George Emil Palade of Targu Mures, 540142 Targu Mures, Romania; 2 First Department of Psychiatry, Clinical County Hospital of Targu Mures, 540142 Targu Mures, Romania; 3 Second Department of Psychiatry, Clinical County Hospital of Targu Mures, 540142 Targu Mures, Romania; 4 Pediatric Cardiology Department, Institute of Cardiovascular Diseases and Cardiac Transplantation of Targu Mures, 540136 Targu Mures, Romania; 5 Department M4, Discipline of Psychiatry, University of Medicine and Pharmacy, Sciences and Technology George Emil Palade of Targu Mures, 540142 Targu Mures, Romania

**Keywords:** pediatric inflammatory bowel disease, psychiatric comorbidities, gut–brain axis

## Abstract

Inflammatory bowel disease (IBD), which includes Crohn’s disease, ulcerative colitis, and unspecified inflammatory bowel disease, is a chronic, unpredictable and immune-mediated condition of the gastrointestinal tract. In pediatric populations, the diagnosis of a chronic and debilitating pathology significantly reduces quality of life. Children diagnosed with IBD may cope with physical symptoms such as abdominal pain or fatigue, but mental and emotional well-being are also important for preventing and reducing the risk of developing psychiatric conditions. Short stature, growth delay and delayed puberty can contribute to poor body image and low self-esteem. Furthermore, treatment per se can alter psycho-social functioning due to the side effects of medication and surgical procedures such as colostomy. It is essential to acknowledge and treat early signs and symptoms of psychiatric distress in order to prevent the development of serious psychiatric disorders in adult life. The literature underlines the importance of incorporating psychological and mental health services as part of the management of inflammatory bowel disease. Diagnosing mental health problems in pediatric patients with IBD can improve their adherence to treatment and pathology course and, consequently, reduce long-term morbidity and mortality.

## 1. Introduction

Inflammatory bowel disease (IBD) is a group of chronic inflammatory conditions of the gastrointestinal system with a remitting–relapsing evolutive character. Crohn’s disease (CD), ulcerative colitis (UC) and unspecified IBD are subtypes described in the literature [1]. Patients frequently experience symptoms such as abdominal pain, diarrhea, weight loss, and delayed growth or fatigue [2,3]. The presenting symptoms of pediatric IBD are summarized in Table 1.

Inflammatory bowel disease is often associated with extraintestinal manifestations (EIM) and complications (EIC) [5]. Reportedly, EIM occur in approximately 5–50% of patients [5]. The organs most commonly affected are joints, skin, ocular system and hepatobiliary tract, although nearly every system may be involved [6]. Table 2 summarizes the extraintestinal manifestations and complications (EIM and EIC) of IBD.

The burden of living with various and multisystemic symptoms, and the need for implementing treatment has piqued considerable interest in the mental health management of patients with IBD [28].

The incidence of inflammatory bowel disease (IBD) appears to be more common in northern countries as well as in industrialized areas [29], and studies have shown that the risk for serious mental illness is generally higher in cities compared to rural areas [30]. Approximately 25% of patients diagnosed with IBD are younger than 18 years old. Although pediatric incidence is currently lower than that in adults, it continues to increase, and there is evidence that the illness can be chronic and has a very aggressive evolution in this particular group of patients [1,31,32].

## 2. Psychological Implications in Inflammatory Bowel Disease

### 2.1. Inflammatory Bowel Disease and Its Psychological Implications in Childhood and Young Adulthood

The pediatric manifestation of inflammatory bowel disease cannot be separated from its psychosocial context. Children and adolescents frequently feel embarrassed and ashamed by fecal incontinence and frequent bathroom visits and often experience social anxiety due to school absences, nutritional strategies such as exclusion diets and distorted body image perception related to short stature or other physical effects of gastrointestinal diseases [33,34]. Additional factors such as age, social support network, primarily represented by the family and coping mechanisms to stress may also influence how adolescents manage and react to their somatic burden [35]. Fatigue is a common symptom reported by children affected by IBD [36] and it refers to a subjectively overwhelming sense of tiredness associated with lack of energy and exhaustion that decreases the capacity for physical and mental activity, decreasing quality of life in similar ways as rheumatologic disease and cancer [37,38]. Fatigue, exhaustion, diminished physical activity and trouble sleeping are more common in children and adolescents with IBD than in their healthy counterparts. Fatigue is likely to be a multifactorial phenomenon and includes biological factors (such as disease activity), psycho-behavioral factors (such as anxiety, depression and family support) disorder [39]. The above-described factors can contribute to significant psychological and functional factors (such as decreased functional capacity) [37,38]. Moreover, fatigue represents a core symptom in major depressive burden that influences disease outcomes and patient’s psychological development [40].

Moreover, inflammatory bowel disease is increasingly being recognized as a complex multifactorial and multisystemic disorder [6]. Alarmingly high rates of depression within the adolescent IBD population have been observed in the largest population-based study, which aimed to evaluate the burden of psychiatric disorders in children and young adults with IBD in USA, comprising a total of 11,316,450 patients aged between 5 and 24 years, including 58.020 patients with a diagnosis of IBD. The prevalence of psychiatric disorders was found to be 21.6% among IBD, and mainly comprised depression and anxiety disorders. The study’s results also indicate that IBD is five times more likely to be associated with psychiatric disorders than controls (*p* < 0.001) [41]. Furthermore, studies on pediatric and adult populations show a significant relationship between depression and anxiety with IBD disease activity and evolution [42,43,44]. In 2018, Van den Brink et al. conducted a randomized controlled trial, concluding that active disease was a significant risk factor for depressive symptoms (OR 4.6, *p* < 0.001). Other significant risk factors for anxiety and/or depression included female gender (OR 1.7), active disease (OR 1.9), and a shorter disease duration (OR 1.4) (all *p* < 0.025) [43]. Furthermore, a meta-analytic review comprising a total of 1167 young people with IBD (M age = 14.33, 50% female) concluded that they had higher rates of depressive disorders and internalizing disorders than young people with other chronic conditions [45]. Extensive research has explored the idea that mental health comorbidities are only a direct result of the global burden of living with a chronic disease or complex mechanism involved in gut–brain interactions. Recent studies indicate that the relationship between IBD and mental illness can be bidirectional, evoking the systemic inflammation that damages the body’s immune and nervous systems and is a possible mechanism for both IBD and mental illness, as well as complex gut–brain interactions [46,47,48,49]. Moreover, another management impairment is that patients with IBD and co-morbid depression and/or anxiety may be less likely to adhere to medical care procedures and therapeutic schemes, resulting in unnecessary escalation in therapy and complications [50,51,52]. The importance of family support and the coordination of the medical treatment are factors of pediatric IBD management that can impact psychological outcomes for children affected by the disease [53]. Younger and middle-aged adolescents benefitted from family support in the context of IBD care management according to Feldman et al.’s (2020) analysis conducted on 76 young IBD patients aged between 11 and 18 [51]. Dealing with a chronic illness and a lifelong management can be overwhelming for children [54]. As described in other chronic disease such as diabetes, poor treatment adherence can cause a high prevalence of psychiatric comorbidities [55]. Lifestyle changes sometimes occur unexpectedly due to disease exacerbation, impacting the lives of both patient and parents, resulting in disruptions to family life. According to a study comprising 87 patients and their parents, a worse disease course was directly associated with the increased distress of parents and indirectly with the lower health-related quality of life (HRQOL) of pediatric patients with IBD, emphasizing that parental distress should be considered in the management of pediatric IBD to improve HRQOL of children [56,57].

Anxiety symptoms may also be related to arising concerns about different lifestyles compared to other children and related to the unknown progression of the illness (relapse or complications of disease requiring surgical procedures or colostomy that can impact one’s body-image perception and self-esteem) [34,58]. Social impairments may be caused by educational deficiencies due to school absences [59,60], as well as low self-esteem due to delayed development or a lack of satisfaction with one’s physical appearance [61]. It is also important to acknowledge and prevent any educational impairments of children with IBD in order to prevent any delays in educational acquisitions. The financial burden of a chronic illness as well as familial sacrifices, repeated hospitalizations, and family issues are problems that a child with IBD may face [62].

Assessing the adaptation of pediatric patients to disease is an important component of long-term management of children with IBD. Existing models of assessment tools focus on evaluating a patient’s social support network, coping mechanisms and skills, daily activities, and body image perception [56,57].

### 2.2. Critic Period: Transition from Pediatric to Adult Inflammatory Bowel Disease Care

While the pediatric management of chronic illnesses includes the benefit provided by the patients’ family support, the transition from children to adult life mandates the development of abilities of self-management and self-care. This transition can be facilitated by gradually increasing the patient’s responsibility and participation in their care plan. Older adolescents should be taught to recognize the warning signs of an emergency and be able to appropriately describe the manifestations and seek immediate treatment, as well as being familiar with administered medication. It is well documented that any discontinuity in patients with IBD care during the transition from a pediatric to adult patient can result in severe adverse outcomes, unfavorable evolution and higher medical costs [53]. Because most patients with pediatric-onset inflammatory bowel disease progress into adulthood, the chances of disease’s long- and short-term complications increase, and burdens of IBD are also increased by psychosocial factors including poor coping ability and other obstacles regarding medical management, as described above. The economic burden and financial impact of a chronic illness must be taken into account in patients experiencing chronic and debilitating symptoms, especially regarding the current evidence for an impaired quality of working life in IBD patients [63]. Regarding work impairments, concentration problems (72%), low working pace (78%) and delayed work productivity (50%) were the most prevalent IBD-related work difficulties described in a questionnaire-based study including 202 IBD patients [64].

It is ideal for the transition from pediatric to adult care to happen when young adults already have a basic understanding of IBD, as well as its therapeutic schemes, prognosis and evolution in both the presence and absence of specific treatments [65,66]. Somatic disease course is beneficial for young adults who have stable mental health, sufficient self-efficacy and are willing to communicate regarding self-care behaviors, developing a beneficial patient–healthcare worker relationship [66,67]. On the other hand, it is widely known that self-management skills may be undermined by the onset of mental illnesses or other psychosocial problems; therefore, it is critical to efficiently diagnose and treat any mental-health-related disturbances.

Findings from a pilot study conducted in the United States of America aimed to understand disease transitions from a broader psychosocial perspective. The results show that transition barriers include disease uncertainty and lack of control, psychological distress, and disruptions to daily life [33]. Facilitators such as mental health support, adequate social support and adequate communication ease the transition process for young patients. Furthermore, the study highlights certain particular and adaptable interventions for facilitating the transition to adulthood IBD care and management, as presented in Table 3 [33,68,69,70].

Although we present several interventions/facilitators, additional research is needed to evaluate the impact of incorporating these exercises into psychosocial programs and to discover further targeted interventions designed to facilitate the transition process.

Finally, these interventions can be introduced to patients at any age and in any order following the opinion of a healthcare professional regarding the most appropriate treatment for the patient and their particularities. However, if not previously initiated, the pediatric-to-adult transition may benefit from the inclusion of these interventions in care management.

The above-mentioned interventions are recommendations that can be implemented during the pediatric IBD care management or as part of transitioning process. The interventions are based on positive psychology and cognitive–behavioral principles, which may be associated with improved disease outcomes across the spectrum of digestive disorders [71].

These facilitating interventions can be introduced to patients at any age and include the following recommendations:

- Develop a disease narrative to develop ways to talk about IBD with those who are not familiar with the disease. Before transitioning to adult care, pediatric patients may benefit from establishing their own disease narrative. By discussing how the disease has affected the patient’s life experiences in a positive way can encourage patients to adopt an optimistic point of view, be self-efficient, and advocate for themselves. This method of communication strengthens mechanisms of growth and learning abilities and was previously associated with happiness, gratefulness, as well as reduced stress and depressive symptoms [72].
- Practicing gratitude has the benefit of enhancing support and strengthening existing connections and social support networks. Examples of gratitude activities include writing letters (in which the patient expresses their gratitude to the persons that helped them during difficult times) and writing a gratitude journal (logging disease symptoms and subsequent emotions that can be easily accessed to help patients focus on the positive aspects of their lives) [33,73].
- Paying it forward means to engage in IBD community activities such as volunteering and mentorship for the newly diagnosed patients. These interventions can boost self-efficacy and are associated with increased satisfaction, well-being and happiness [74].
- Setting SMART goals is a means to divide up large and sometimes difficult goals into smaller and more achievable targets. Setting SMART goals for IBD management can allow adolescent patients and their caregivers to prioritize the goals of interest, focusing on optimizing their chances of successfully completing the tasks, consequently building efficacy that prepares patients to reach the disease management independency.

SMART is an acronym that highlights the key features of the goal:

- Specific: what, where, and when will we work toward on this goal?
- Measurable: how can we evaluate if we have achieved the goal or not?
- Achievable: is this goal within the realm of possibility?
- Relevant to larger goals: does this goal fit into my value system?
- Time-sensitive: at what point will we check in on progress to determine whether we have reached the goal? [75]
- Mastering the gut–brain axis means to optimize education about the complex interactions of the gut–brain axis that may help pediatric patients to better manage their disease. Helping patients differentiate between their symptoms can help reduce concerns for those who may feel that stress-induced symptoms are not important. It is therefore mandatory for doctors to identify certain concerning symptoms and establish multidisciplinary approaches with qualified mental health professionals (psychologists and psychiatrists) [33,76].

### 2.3. Anatomopathology and Reported Pathogenesis of Psychiatric Implications in Patients with Inflammatory Bowel Disease

The complexity of the gut–brain axis has sparked significant interest from researchers over the last twenty years [49,77,78]. The gut–brain axis is a complex bidirectional communication system driven by neural, hormonal, metabolic, immunological, and microbial signals [77,78]. Signaling events from the gut can modulate brain function, and recent evidence suggests that the gut–brain axis may play a pivotal role in linking gastrointestinal and neuropsychiatric pathologies [78,79]. In a review article, RK Masanetz et al. explored a series of cascade events along the gut–immune–brain axis, initiated by the evasion of chronic intestinal inflammation, that pass through the epithelial–vascular barrier in the gut and cause systemic inflammation [79]. Moreover, the malfunctioning of the gut–brain axis described in IBD patients includes structural and functional abnormalities of the enteric nervous system (ENS) and induced abnormal, innate, and adaptive immunological responses, affecting intestinal barrier integrity and leading to systemic fallout [80]. It has been postulated that neuroinflammation-induced depression in IBD involves peripheral inflammatory mediators originating from the inflammation in the gastrointestinal system, penetrating the blood–brain barrier and activating the resident macrophage-like microglial cells within the central nervous system [46,81,82].

It has been reported that neuroinflammation can induce the dysregulation of the hypothalamus–pituitary–adrenal (HPA) axis [83] and decrease serotonin levels [84], as well as altering the hippocampus [85], mechanisms involved in major depressive disorder (MDD) [81]. In a previous study, low serum serotonin levels were correlated with depressive symptoms [86] and in a small study, plasmatic serotonin level was used as a screening marker for anxiety and depression in patients with type 2 diabetes [87]. Further studies are essential for evaluating the use of serum serotonin as a predictor of depression.

Bonaz BL (2013) suggested a dysfunction of brain–gut interactions in the pathogenesis of IBD, evidenced by the dysfunctionality of the autonomic nervous system and abnormal functioning of the hypothalamic–pituitary–adrenal axis. Their study is supported by [77], which found similar results for the cholinergic anti-inflammatory pathway [88]. The harmful effect of stress, abnormal association of the prefrontal cortex–amygdala, and an abnormal microbiota–brain relationship were found in pro-inflammatory models [83,88].

In Neurogastroenterology and Motility, Agostini et al. (2013) evaluated patients with Crohn’s disease for brain volumetric alterations, and the results indicate decreased gray matter volumes in the dorsolateral prefrontal cortex and anterior midcingulate cortex. Illness time evolution was negatively correlated with volumetric measures in the subgenual anterior cingulate cortex, posterior midcingulate cortex, ventral posterior cingulate, and parahippocampal cortex [89]. The anterior midcingulate cortex was responsible for feedback-mediated decision making [90]. The gray matter volume in the subgenual anterior cingulate cortex was found to be abnormally reduced in subjects with major depressive disorder and bipolar disorder [91,92]. The ventral posterior cingulate cortex plays a role in self-monitoring the personal relevance of somatosensory stimuli. It is also involved in spatial processing, actions in space, and some types of memory pathways [93] that may be interrupted by posterior cingulate cortex atrophy, as discovered in small studies of Crohn’s disease [89,94]. Moreover, a systematic multi-database study comparing adult IBD patients versus healthy controls (*n* = 687) concluded that IBD patients had significant deficits in attention, executive function, and working memory compared with healthy controls, suggesting that cognitive impairment is a potential extraintestinal manifestation of IBD [95]. Another small comparative study on a pediatric population found that patients with IBD and cystic fibrosis performed more poorly than healthy controls in attention and memory tests [96].

Moreover, white matter alterations have also been observed in IBD patients compared to controls in a small number of studies on nervous system imaging in patients with IBD. The lesions were clinically asymptomatic and potentially associated with IBD, and whether these structural changes represent a unique extraintestinal manifestation of the disease remains unclear and further studies must be conducted [97]. In contrast, a study with a relatively small sample size did not find an increased rate of white matter lesions among patients with IBD when compared to healthy controls [98]. Conflicting data and small studies regarding white matter lesions mandate further studies.

Inflammatory bowel disease has been associated with a higher risk of cerebrovascular accidents [99] according to a study including 261,890 IBD patients compared to non-IBD patients (6.24% vs. 0.48%, *p* < 0.0001) [100]. The hypercoagulability [14] state present in IBD could be a mechanism by which the presence of IBD might lead to the development of cerebrovascular accidents.

### 2.4. Mental Disorders Associated with Inflammatory Bowel Disease

In patients with IBD, several studies have described an increased frequency of various mental disorders, ranging from mild depression to severe forms of schizophrenia and serious dementia, but the prevalence of mental disorders depends on the study, as described below.

Upon examining the literature, depression and anxiety disorders were the most studied mental health disorders, both in pediatric and adult IBD patients [41,101,102].

IBD patients often suffer from anxiety and depression that may influence the course of disease via the complexity of the gut–brain axis [101]. In a systematic review, Mickoka et al. (2016) demonstrated that the prevalence of anxiety and depression is higher in patients with IBD and even higher in the active phase of the disease [101]. A review and meta-analysis of 60.114 adult and pediatric IBD patients identified high prevalence of: mood disorders, 10%; anxiety disorders, 12%; substance misuse, 3%; psychotic disorders, 2%; behavioral disorders, 1%; personality disorders, 3%; developmental disorders, 1%; and behavioral and emotional disorders with onset usually during childhood, 1% [102].

Thavamani et al. conducted a retrospective case–control analysis, including 58,020 IBD pediatric patients, of which 12,540 were diagnosed with psychiatric disorders. Depression, anxiety and adjustment disorder contributed to about 95% of all psychiatric disorders. Among the screened psychiatric disorders, IBD patients had an increased risk of associated anxiety, depression, panic disorder, phobias and dysthymias [41]. The study results are presented in Table 4.

Comparatively, Bernstain et al. conducted a retrospective matched-control study analyzing the incidence and prevalence of psychiatric disorders in an adult IBD cohort compared with a matched cohort without IBD [103]. The results are presented in Table 5.

Various researchers studied the risk factors of depression among patients with IBD and concluded that age, severe disease, flare ups, disability, unemployment, socioeconomic impairment [104] and coronavirus disease 2019 isolation due to extensive lockdown measures are factors responsible for developing depressive symptoms [105]. Various studies highlight the increased risk of suicide among IBD patients [106,107,108].

There is conflicting research data regarding the association between bipolar disorder and inflammatory bowel disease. A population-based, cross-sectional study from Taiwan demonstrated that patients with IBD were more likely to have bipolar disorder. Moreover, a national cohort study conducted in Denmark presented a higher risk of bipolar disorder only in patients with CD [109,110]. Thavamani et al. observed a high odds ratio (OR) of bipolar disorder in an IBD pediatric population [41]. In 2022, Wang et al. found a significant, positive association for genetically predicted bipolar disorder with the risk for IBD and ulcerative colitis (per log/odds ratio increase; odds ratios: 1.18 and 1.19, respectively) [111].

Regarding comorbid IBD–schizophrenia, studies show inconsistent results. While a population-based cohort study conducted in Taiwan (2020) demonstrated a significant association between schizophrenia and subsequent IBD development (1.14% vs. 0.25% in non-IBD population), other studies failed to demonstrate any association between somatic and mental illnesses [112,113]. In pediatric populations, rare and isolated cases of medication-induced psychosis are described in the literature [114,115]

Recent evidence suggests there is a possible association between IBD and eating disorders, although the exact mechanisms involved in its ethio-pathogenesis are not fully understood. This association may lead to worse prognosis [108,116,117].

Table 6 presents the association between childhood-onset inflammatory bowel disease and mental health disorders in adulthood as well as the specific adaptation hazard ratios as found by Butwicka et al. (2019) [108].

Moreover, suicide represents a serious global public health burden. According to the WHO (World Health Organization), approximately 703,000 people per year die from suicide worldwide [118]. In 2019, suicide accounted for more than 1 out of 100 deaths, and 58% of suicides occurred before age 50. Mental disorders are the leading cause of disability worldwide, accounting for one in six cases of disability [119]. Patients diagnosed with severe mental health conditions die on average 10–20 years earlier than the general population. Sexual molesting and bullying are major causes of depression in children, and bullying should not be overlooked in pediatric IBD patients, especially in those who lack confidence in their physical appearance [119].

The most severe outcome in patients with depression is suicide, but the risk of suicide in patients with IBD is not entirely clear. A systematic review based on 28 studies concluded that patients with inflammatory bowel disease were associated with an increased risk of suicide attempts (relative risk= 1.39; 95% CI, 1.08–1.79) and deaths from suicide (relative risk= 1.25; 95% CI, 1.09–1.43) compared to controls. The review also concluded that Crohn’s disease subtypes, female patients, childhood-onset IBD, young-adult IBD, and short-duration IBD characteristics are factors that carry a high risk of suicide [106]. Another case–control study regarding the Danish population highlights the necessity to correctly assess the risk of suicide among IBD patients. The results indicate increased rates of suicide among participants with CD (odds ratio = 1.6, 95%) and UC (OR = 1.9, 95%) [120]. Butwicka et al. observed an increased hazard ratio (HR = 1.2, respectively 1.5) in UC and childhood-onset CD [108].

Regarding these findings, healthcare providers should consider a multidisciplinary approach that includes medical and psychological factors. Psychological factors should always be considered via assessments of depressive symptomology and the co-occurrence of suicide risk, especially if patients with IBD present co-occurring factors, such as active disease phase or high pain levels, that increase the risk of suicide [59]. The identification and treatment of mental health conditions such as depression in IBD populations is essential for reducing the high number of patients at risk of suicide [121]. Childhood traumas must also be assessed, as certain traumatic events may increase suicide risk [122].

## 3. Conclusions

To our knowledge, childhood-onset inflammatory bowel disease has an impact on more than just the gastrointestinal tract; patients often have psychological comorbidities and mental health implications further in adult life. Although the pathogenetic mechanisms between inflammatory bowel disease and mental illnesses are not fully elucidated, genetic factors and the gut–brain interaction seem to contribute to the increased prevalence of mental disorders in patients with IBD, and further studies must be established in order to describe and elucidate the complex mechanisms. Childhood-onset inflammatory bowel disease tends to have a more severe evolutive course than IBD diagnosed in adulthood, and mental health burden adds to the overall low quality of life rates described in this particular group of patients. Psychiatric comorbidities are independent predictors of the severity of IBD symptoms, not only negatively impacting a patient’s quality of life, but also increasing healthcare utilization and economic burden. The importance of family support and the coordination of the medical treatment is a particularity that can impact psychological outcomes for children affected by inflammatory bowel disease. Addressing mental health problems in pediatric patients with IBD can improve their medication adherence and somatic disease course, reducing overall morbidity and mortality. Specific measurable variables such as serum serotonin levels may become useful tools in assessing a patients’ risk of developing psychiatric comorbidities such as depression, but further larger research studies are required.

Understanding the need to implement various transition mechanisms of psychological support from childhood to adulthood care in patients with IBD is important for both healthcare providers and insurance payers, as it has a direct impact on medical care and disease outcomes. It is ideal for the transition from child to adult care to happen when young adults already have a good general understanding of their disease. Social and educational impairments in pediatric patients require special attention regarding the management of inflammatory bowel disease patients because adaptation difficulties can underlie the development of serious mental health problems in adult life and decrease the global functionality.

Somatic disease courses may benefit patients who are stable in terms of their mental health and have sufficient self-efficacy and willingness to communicate regarding self-care behaviors.

The increased frequency of psychiatric comorbidities in childhood-onset inflammatory bowel disease, as described in the literature, especially mood and anxiety disorders, demonstrates that early interventions can prevent and attenuate the development of serious psychiatric pathologies; however, further studies and investigations are mandatory for developing the best approaches and multidisciplinary protocols to manage mental health implications in pediatric inflammatory bowel disease. We believe that further prospective follow-up studies on the prevalence of psychiatric disorders in childhood-onset IBD populations would be greatly valuable and of considerable interest to researchers.

## Figures and Tables

**Table 1:** Frequent presenting symptoms in pediatric IBD [4].

General	Gastrointestinal Tract
Weight loss	Abdominal pain
Fever	Diarrhea
Anorexia	Rectal bleeding
Delayed growth	Nausea/vomiting
Lethargy/fatigue	Constipation
	Perianal disease (CD)
	Mouth ulcers

**Table 2:** Extraintestinal manifestations (EIM) and complications (EIC) of IBD.

System	Manifestation/Complication
Generalized	Fever [7], weight loss [8], fatigue [7,9], nausea/vomiting/appetite changing [7]
Ocular	Uveitis/episcleritis/iritis/conjunctivitis [6,10]
Oral	Cheilitis/stomatitis/oral ulcerations [6,11]
Pulmonary	Pulmonary vasculitis/fibrosing alveolitis [12,13]
Vascular	Vasculitis/thrombosis [14,15]
Hepatobiliary	Primary sclerosing cholangitis/fatty liver disease/granulomatous hepatitis/autoimmune liver disease/cholestasis/gallstone formation [6,16]
Pancreatic	Pancreatitis (acute, chronic, autoimmune) [17]
Renal/Urinary	Nephrolithiasis [18]/tubulointerstitial nephritis [19]/glomerulonephritis [20]/amyloidosis [21]
Hematologic	Iron deficiency/chronic anemia/thrombocytosis/vitamin B12 deficiency/autoimmune hemolytic anemia [6,22]
Endocrine	Decreased growth velocity/delayed sexual maturation [23,24]
Integumentary	Erythema nodosum/pyoderma gangrenosum/perianal disease/CD [6,25]
Musculoskeletal Neuropsychiatric	Osteopenia and osteoporosis/arthritis/arthralgias/ankylosing spondylitis/sacroiliitis [23,26] Venous and arterial thrombotic and thromboembolic events/demyelinating diseases/peripheral neuropathies/white matter lesion/psychiatric disorders [27]

**Table 3:** Interventions considered to facilitate IBD care transition [33].

Adaptable Intervention	Elements/Examples		
Develop a disease narrative	What is IBD and what symptoms are most prominent for you day-to-day? What are some of the challenges that you might experience related to having IBD (e.g., managing medications, medical procedures, dietary or activity restrictions)? In what ways have you grown through the challenges of living with IBD?		
Practice gratitude	Write a gratitude letter Keep a gratitude journal		
Pay it forward	Engagement with the IBD community Voluntary Mentorship		
Set SMART Goals	Specific Measurable Achievable Relevant Time-sensitive	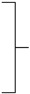	goals
Master the gut–brain axis	Optimizing education about the complex interactions of the gut and brain axis Understanding relationship stress symptoms Useful questions: What do you notice about how your stress levels influence your IBD symptoms? Conversely, when you begin to experience the onset of IBD symptoms, how does that impact your mental state?		

**Table 4:** Association between psychiatric disorders and inflammatory bowel disease in pediatric population and young adults (<24 years) (adapted following Thavamani et al.) [41].

Mental Health Disorder	Odds Ratio (OR)	Lower CI-Upper CI	*p*-Value
Bipolar disorder	3.5	3.3–3.73	<0.001
Depression	3.94	3.82–4.06	<0.001
Anxiety disorder	4.05	3.96–4.16	<0.001
Any mental health disorder	3.83	3.75–3.91	<0.001

**Table 5:** Association between psychiatric disorders and inflammatory bowel disease in young adult (18–24 years) and adult populations (>24 years) (adapted following [103]).

Mental Health Disorder	IRR Incidence Rate Ratio in IBD Patients	IRR in Match Cohort	*p* Value
Depression	1.58 (>)	1.0	*p* < 0.05
Anxiety disorder	1.39 (>)	1.0	*p* < 0.05
Bipolar disorder	1.82 (>)	1.0	*p* < 0.05
Schizophrenia	1.64 (>)	1.0	*p* > 0.05

**Table 6:** Association between childhood-onset IBD and psychiatric comorbidities [108].

Psychiatric Pathology in Patients with Childhood-Onset Inflammatory Bowel Disease						
Psychiatric Pathology	Ulcerative Colitis		Crohn′s Disease		IBD—Unclassified	
	HR (85% CI)	*p* Value	HR (85% CI)	*p* Value	HR (85% CI)	*p* Value
Psychotic disorders	0.9 (0.5–1.5)	0.58	1.3 (0.8–2.2)	0.27	0.8 (0.2–3.1)	0.7
Mood disorder	1.4 (1.3–1.7)	<0.001	1.6 (1.4–1.9)	<0.001	1.8 (1.3–2.4)	<0.001
Anxiety disorders	1.6 (1.4–1.8)	<0.001	2.2 (1.9–2.4)	<0.001	2.4 (1.9–3.0)	<0.001
Eating disorders	1.3 (0.9–1.8)	0.13	1.9 (1.4–2.6)	<0.001	2.4 (1.3–4.3)	0.005
Substance misuse	1.1 (0.9–1.3)	0.33	1.2 (0.9–1.4)	0.16	0.7 (0.4–1.1)	0.12
Personality disorders	1.4 (1.0–2.0)	0.03	1.2 (0.8–1.8)	0.44	2.0 (1.0–3.9)	0.04
Behavioral disorders	0.8 (0.3–1.9)	0.64	2.0 (1.0–3.9)	<0.05	1.4 (0.3–5.8)	0.62
ADHD	1.2 (1.0–1.5)	0.07	1.2 (0.9–1.5)	0.13	1.1 (0.6–1.7)	0.8
Autism spectrum disorders	1.5 (1.1–2.1)	0.01	1.2 (0.8–1.7)	0.42	1.6 (0.8–3.3)	0.19
Intellectual disability	1.1 (0.7–1.9)	0.62	0.9 (0.4–1.7)	0.68	2.7 (1.2–5.7)	0.01
All psychiatric disorders	1.4 (1.3–1.5)	<0.001	1.7 (1.6–1.9)	<0.001	1.8 (1.5–2.1)	<0.001
Suicide attempts	1.2 (0.9–1.6)	0.23	1.5 (1.1–2.0)	0.01	2.3 (1.4–3.8)	0.001

Abbreviations: HR—hazard ratio, IBD—inflammatory bowel disease.

## Data Availability

Not applicable.

## Abbreviations

CD	Crohn’s disease
CI	Confidence interval
EIM	Extraintestinal manifestations
EIC	Extraintestinal complications
HPA	Hypothalamus–pituitary–adrenal axis
HR	Hazard ratio
HRQOL	Health-related quality of life
IBD	Inflammatory bowel disease
MDD	Major depressive disorder
OR	Odds ratio
IRR	Incidence rate ratio
UC	Ulcerative colitis
WHO	World Health Organization
